# Evaluation of the participation of *ABCA1* transporter in epicardial and mediastinal adipose tissue from patients with coronary artery disease

**DOI:** 10.20945/2359-4292-2023-0188

**Published:** 2023-12-01

**Authors:** Giovanny Fuentevilla-Álvarez, Claudia Huesca-Gómez, Yazmín Estela Paz-Torres, Nadia González-Moyotl, María Elena Soto, José Antonio García-Valdivia, Reyna-Sámano, Martín Martínez-Rosas, Sergio Enrique Meza-Toledo, Ricardo Gamboa

**Affiliations:** 1 Instituto Nacional de Cardiología “Ignacio Chávez” Departamento de Fisiología Ciudad de México México Departamento de Fisiología, Instituto Nacional de Cardiología “Ignacio Chávez”, Ciudad de México, México; 2 Instituto Politécnico Nacional Escuela Nacional de Ciencias Biológicas Departamento de Bioquímica Ciudad de México México Departamento de Bioquímica, Escuela Nacional de Ciencias Biológicas, Instituto Politécnico Nacional (IPN), Ciudad de México, México; 3 Instituto Nacional de Cardiología “Ignacio Chávez” Departamento de Inmunología Ciudad de México México Departamento de Inmunología, Instituto Nacional de Cardiología “Ignacio Chávez”, Ciudad de México, México; 4 I.A.P.A.B.C. Cardiovascular Line in American British Cowdray (ABC) Medical Center Mexico City Mexico Cardiovascular Line in American British Cowdray (ABC) Medical Center, I.A.P. A.B.C., Mexico City, Mexico; 5 Instituto Nacional de Perinatología Coordinación de Nutrición y Bioprogramación Ciudad de México México Coordinación de Nutrición y Bioprogramación, Instituto Nacional de Perinatología, Ciudad de México, México

**Keywords:** Coronary heart disease, ABCA1, single nucleotide polymorphism, mRNA expression

## Abstract

**Objective:**

Recent studies have shown a relationship between adipose tissue and coronary artery disease (CAD). The ABCA1 transporter regulates cellular cholesterol content and reverses cholesterol transport. The aim of this study was to determine the relationship between single nucleotide polymorphisms (SNPs) R230C, C-17G, and C-69T and their expression in epicardial and mediastinal adipose tissue in Mexican patients with CAD.

**Subjects and methods:**

The study included 71 patients with CAD and a control group consisting of 64 patients who underwent heart valve replacement. SNPs were determined using TaqMan probes. mRNA was extracted using TriPure Isolation from epicardial and mediastinal adipose tissue. Quantification and expression analyses were done using RT-qPCR.

**Results:**

R230C showed a higher frequency of the GG genotype in the CAD group (70.4%) than the control group (57.8%) [OR 0.34, 95% CI (0.14-0.82) p = 0.014]. Similarly, C-17G (rs2740483) showed a statistically significant difference in the CC genotype in the CAD group (63.3%) in comparison to the controls (28.1%) [OR 4.42, 95% CI (2.13-9.16), p = 0.001]. mRNA expression in SNP R230C showed statistically significant overexpression in the AA genotype compared to the GG genotype in CAD patients [11.01 (4.31-15.24) vs. 3.86 (2.47-12.50), p = 0.015].

**Conclusion:**

The results suggest that the GG genotype of R230C and CC genotype of C-17G are strongly associated with the development of CAD in Mexican patients. In addition, under-expression of mRNA in the GG genotype in R230C is associated with patients undergoing revascularization.

## INTRODUCTION

Cardiovascular diseases are a group of disorders of the heart and blood vessels (1), and one of the most common worldwide is coronary artery disease (CAD). Fat accumulation around the heart has been established as a risk factor for various cardiovascular diseases. Accumulation in the cardiac region occurs inside and outside the pericardium in fatty deposits in the epicardial and pericardial areas (essentially, mediastinal adipose tissue [MAT]). Epicardial adipose tissue (EAT) has been the target of various investigations due to its relationship with the coronary arteries, the myocardium, and its potential influence on CAD development. EAT comprises visceral fat below the visceral pericardium and is in close contact with the coronary arteries (2).

Studies have reported that inflammation regulates ATP-binding cassette transporter A1 (*ABCA1*) as its expression is suppressed by proinflammatory cytokines (3,4). ABCA1 is a membrane transporter with a primary function of regulating the efflux of cholesterol and phospholipids to lipid-poor apoA1, which is an HDL precursor (5) that is widely expressed in fat tissue. Furthermore, adipose tissue lipolysis is associated with ABCA1 activity (6). ABCA1 mediates the rate-limiting step of HDL biogenesis, which is essential in cholesterol homeostasis and reverse cholesterol transport (RCT) (7). The *ABCA1* gene is a highly polymorphic gene that is located on human chromosome 9 (9q31.1). It has a length of 149 kb and 50 exons that encode integral membrane protein with a size of 240 kDa and 2,261 amino acids (8).

Among healthy people and those with coronary heart disease (CHD), the most common *ABCA1* gene mutations are single nucleotide polymorphisms (SNPs). DNA sequence polymorphisms caused by single nucleotide variation is the most common type of human genetic variation and accounts for more than 90% of known polymorphisms (9). More than 5,000 polymorphisms have been reported in this gene or near it, and several of these variants (intronic, missense, and located in the promoter region) affect the expression and function of the ABCA1 protein. Both rare and common genetic variations in *ABCA1* contribute to circulating levels of HDL cholesterol in population-based studies (6). To gain insight into the regulatory mechanisms involved in *ABCA1* gene expression, our objective was to determine the relationship between SNPs R230C, C-17G, and C-69T and their mRNA expression in EAT and MAT in patients with CAD.

## SUBJECTS AND METHODS

### Patient population

A case-control study was carried out with 135 subjects, including adults over 18 years of any sex who agreed to participate in the study. There were 71 patients with angiographically proven obstructive CAD who underwent elective primary coronary artery bypass surgery, which is defined as a disease that causes stress- or exercise-related symptoms of angina due to a narrowing of ≥50% in the left common trunk or ≥70% in one or more of the major arteries. The control subjects were 64 patients undergoing valve replacement that was unrelated to atherosclerosis lesions and had normal coronary angiography in the preoperative period. Patients were excluded from both groups if they had liver disease, kidney disease, cancer disease, untreated abnormal functioning of the thyroid gland, infectious processes, corticosteroid treatment, non-Mexican ancestry, and contaminated or insufficient samples. All participants answered standardized and validated questionnaires to obtain information on family and medical history, physical activity, and alcohol and tobacco consumption.

### Ethical aspects

Signed informed consent forms were obtained from each participant after fully explaining the purpose and nature of all procedures used in the study in accordance with the Declaration of Helsinki (10). The research was approved by the Ethical, Biosecurity, and Investigation Committees of the National Institute of Cardiology (registration number 10-690).

### Blood samples

Five mL of venous blood were collected in tubes with EDTA and without anticoagulant after 12 h of fasting. Plasma was separated by centrifugation for determination of the lipid profile.

### Laboratory analysis

Total cholesterol, triglycerides, and glucose were determined on a Hitachi 902 autoanalyzer (Böehringer Mannheim, Germany) using commercial enzymatic kits (Roche Diagnostics, Mannheim, Germany and Wako Chemicals, USA). HDL-C was determined by a homogeneous enzymatic method (Roche Diagnostics, Mannheim, Germany).

### DNA extraction

Genomic DNA was isolated from blood samples using a commercial kit (Invitro-gen Co., Carlsband, CA, USA). The DNA was quantified with a spectrophotometer (BioPhotometer plus) at wavelengths of 260/280 nm.

### Determination of polymorphisms

Polymorphisms were selected after considering published reports (Table 1). The frequencies reported in other populations were reviewed in HapMap. The different polymorphisms were determined using TaqMan probes with the CFX96TM Touch Real-Time PCR Detection System. Applied Biosystems synthesized the probes. To this end, 6 μL of TaqManTM Universal PCR Master Mix were used in a reaction volume of 10 μL with final concentrations of 10 ng/μL of DNA, 700 nM of primers, and 100 nM of the probe labeled with fluorophores.

**Table 1:** Information on the studied SNPs

Gene SNP	*ABCA1 rs9282541*	*ABCA1 rs2740483*	*ABCA1 rs1800977*
Chromosome	9	9	9
SNP Type	Exon 7 Variant	5’ UTR C>T	5’ UTR C>T
Position	R230C	C-17G	C-69T
MAF	0.04	0.25	0.44

SNPs: single nucleotide polymorphisms; MAF: minor allele frequency. Data obtained from the 1,000 Genomes Project.

The reaction conditions were 10 min at 95 °C, 40 cycles at 92 °C for 15 s, and 1 min at 60 °C. The fluorescence levels of the PCR products were quantified using CFX96 Software (Bio-Rad). To confirm the genotyping data, 80% of samples (including controls and cases) were selected randomly for repeat analysis. The replication rate of data was 100%.

### Epicardial and MAT

EAT and MAT biopsies were obtained during revascularization and valve replacement surgeries. In the case of EAT, biopsies with an average weight of 0.5-1 g were obtained from the region proximal to the left anterior descending coronary artery due to lesions in different segments. Samples of MAT were obtained from the pectoral region. The samples were placed in tubes with Allprotect Tissue Reagent (QIAGEN, Hilden, Germany) and frozen at −70 °C until RNA and protein extraction.

### mRNA extraction and quantification by RT-qPCR

mRNA was extracted using the TriPure Isolation Reagent technique (Roche Molecular Biochemicals, UK). RT-qPCR was then performed, which started with 1 μg of total RNA for cDNA synthesis according to the High-Capacity cDNA Reverse Transcription Kit (Applied Biosystems, Foster, CA, USA). mRNA was quantified using a Bio-Rad CFX96 Real-Time System (Bio-Rad, Hercules, CA, USA). Expression levels of *ABCA1* (Hs00233899_ml) and *HPRT1* (Hs99999909_m1) (reference gene) were measured using a commercially available kit (TaqMan Gene Expression Assay, Applied Biosystems). Amplifications were performed by starting with a 10-minute template denaturation step at 95 °C, followed by 40 cycles at 95 °C for 15 seconds and 60 °C for 1 minute. All analyses were performed in duplicate. Data were expressed relative to each control value, and relative quantification was carried out using the formula 2-ΔΔC (11).

### Statistical analysis

The Hardy-Weinberg equilibrium (HWE) for controls and patients was determined using a chi-squared test. Polymorphism analysis was calculated using SPSS version 18 (SPSS Chicago, Il, USA) and the EPISTAT statistical program (Version 5.0; USD Incorporated 1990, Stone Mountain, Georgia). Pooled odds ratios (ORs) with 95% confidence intervals (CIs) were calculated to assess the associations of the *ABCA1* R230 A/G, C-17G, and C-69T polymorphisms with CAD and the control group, as well as dominant (11 *vs.* 12 + 22 for R230, C-69T, and C-17G), over-dominant (12 *vs.* 11 + 22 for R230, C-69T, and C-17G), recessive (22 *vs.* 12 +11 for R230, C-69T, and C-17G), co-dominant 1 (11 *vs.* 12 for R230, C-69T, and C-17G), co-dominant 2 (11 *vs.* 22 for R230, C-69T, and C-17G), additive (2(22) + 12 *vs.* + 11 for R203, C-69T, and C-17G), and allelic (1 *vs.* 2 for R230, C-69T, and C-17G) models. Statistical significance was defined using p ≤ 0.05.

Logistic regression analyses were performed to assess the associations of ABCA1 with clinical variables under different inheritance models. The p-values were obtained according to the number of comparisons performed. Relative risk was calculated as the odd ratio using 95% CIs. *ABCA1* mRNA expression data are presented as median (minimum-maximum) values. Comparisons between mRNA expression groups were performed using the Mann-Whitney U test for discontinuous variables. A one-way ANOVA was performed to compare the levels of the lipids, glucose profiles, and anthropometric data while stratifying the population by genotype and alleles in both study groups (CAD and control group).

## RESULTS

### Clinical characteristics

A case-control study was conducted with 135 patients, including 71 patients who had CAD and 64 control subjects. Table 2 shows the clinical and anthropometric characteristics and statistical differences between groups. The mean age was 60.28 ± 10.30 years for patients with CAD and 47.83 ± 21.11 years for the control group. However, there were no significant differences when comparing the other variables between groups. In the CAD group, 71.40% used statin drugs, 51.65% used hypoglycemic agents, and 73.8% used antihypertensives.

**Table 2:** Clinical characteristics

Variable	CAD (n = 71)	Controls (n = 64)	p
Age (years)	60.28 ± 10.30	47.83 ± 21.11	<0.001
Sex % M/F	81.7 %/18.3	88.9%/11.1	0.490
BMI (kg/m^2^)	26.33 ± 3.4	26.18 ± 4.02	0.817
TC (mg/dL)	148.38 ± 53.64	137.71 ± 49.16	0.317
HDL-C (mg/dL)	34.13 ± 9.27	30.83 ± 8.42	0.073
LDL-C (mg/dL)	97.41 ± 47.60	81.33 ± 39.81	0.221
Triglycerides (mg/dL)	153.67 ±75.13	154.20 ± 57.69	0.970
Glucose (mg/dL)	121.32 ± 53.04	108.51 ± 35.97	0.247
T2DM (≥126 mg/dL) %	40	12.7	0.002
Hypertension (≥140 mmHg) %	56.5	42.3	0.330
Statins %	71.40	7.9	0.001
Hypoglycemic agents %	8.3	3.0	0.110
Antihypertensive %	61.3	40.9	0.011

The data are expressed as the mean ± SD (Student's t-test).

BMI: body mass index; TC: total cholesterol; HDL-C: high-density lipoprotein cholesterol; LDL-C: low-density lipoprotein cholesterol; SBP: systolic blood pressure; DBP: diastolic blood pressure; T2DM: type 2 diabetes mellitus.

### Genotype and allelic frequencies of *ABCA1* (rs9282541, rs2740483, rs1800977)

The genotype distributions of C-17G and C-69T were in accordance with those predicted by the HWE, but not for R230C (rs9282541) in the control group (χ^2^ = 4.091, p = 0.043) and CAD group (χ^2^ = 29.51, p ≤ 0.001). We analyzed the associations between *ABCA1* genotypes in different inheritance models in patients who did not present an atheromatous process and patients undergoing revascularization surgery. The minor allele frequency (MAF) was considered a reference in constructing inheritance models.

In R230C, the GG genotype had a higher frequency in the CAD group (70.4%) than the control group (57.8%) with a significant difference in the overdominant model [OR: 0.34, 95% CI, 0.14-0.82), p = 0.014]. Similarly, in the case of C-17G, the results showed a statistically significant difference in the CC genotype in the CAD group (63.3%) in comparison to the control group (28.1%) [OR: 4.42, 95% CI, (2.13-9.16), p = 0.001] and allele C (≤0.0001). Finally, for SNP C-69T, there were significant differences in the additive model [OR: 0.37, 95% CI, (0.17-0.81), p = 0.011] (Table 3). A one-way ANOVA was also performed between genotypes, anthropometric data, and lipid profiles to confirm whether any genotype is associated with abnormal lipid and glucose levels. However, no association presented significant differences (data not shown).

**Table 3:** Genotypic and allelic frequencies of *ABCA1* SNP in different models in CAD and controls patients

*ABCA1*	Genotype frequency n (%)			Model	P value	OR (95% CI)
**R230C**	**AA**	**AG**	**GG**			
Control	8 (12.5)	19 (29.6)	37 (57.8)	Dominant	0.47	1.42 (0.54-3.74)
CAD	12 (16.9)	9 (12.6)	50 (70.4)	Overdominant	**0.014**	0.34 (0.14-0.82)
				Recessive	0.12	1.73 (0.85-3.52)
				Codominant	0.10	3.16 (0.95-10.46)
				Codominant 2	0.96	1.10 (0.41-98)
				Additive	0.94	0.94 (0.37-2.48)
				Allelic	0.43	0.80 (0.46-1.13)
**C-17G**	**CC**	**CG**	**GG**			
Control	18 (28.1)	35 (54.7)	11 (17.1)	Dominant	**0.001**	4.42 (2.13-9.16)
CAD	45 (63.3)	25 (35.2)	1 (1.4)	Overdominant	0.023	0.45 (0.22-0.89)
				Recessive	0.003	0.06 (0.08-0.54)
				Codominant	0.001	3.5 (1.65-7.40)
				Codominant 2	0.19	0.51 (0.19-1.39)
				Additive	≤0.0001	0.06 (0.015-0.30)
				Allelic	≤0.0001	3.14 (1.98-5.98)
**C69T**	**AA**	**AG**	**GG**			
Control	11 (17.6)	35 (54.6)	18 (28.1)	Dominant	0.12	1.88 (0.82-4.33)
CAD	20 (28.1)	34 (47.8)	17 (23.9)	Overdominant	0.43	0.76 (0.38-1.49)
				Recessive	0.57	0.80 (0.37-1.73)
				Codominant 1	0.23	1.87(0.78-4.48)
				Codominant 2	0.23	1.87 (0.78-4.48)
				Additive	**0.011**	0.37 (0.17-0.81)
				Allelic	0.21	1.35 (0.83-2.18)

OR: odds ratio.

To evaluate the effect of the genetic charge in different ethnic groups, we compared the allele frequencies of our patients with those of healthy subjects from different populations reported in the literature (Table 4). SNP R230C did not show differences between the prevalence of the alleles when comparing our results to those of indigenous populations of Yaqui and Maya people from Mexico. When comparing our results of the C-17G SNP to those of a Chinese population, we found significant differences in the prevalence of the C allele (our result: 55.4%; Chinese population: 22.6%; p < 0.001). Finally, we compared the alleles of the SNP C-69T to those from Istanbul and Egypt, and the results showed significant differences with both populations (p = 0.002 and p < 0.001 respectively).

**Table 4:** Comparison between alleles frequencies in different populations

Population	*ABCA1* R230C %		p	*ABCA1* C-17G %		p	*ABCA1* C-69T %		p
	A	G		C	G		A	G	
Our study	27.3	72.7	−	55.4	44.5	−	44.5	55.5	−
Yaqui (12)	20.3	79.7	0.41	−	−	−	−	−	−
Maya (12)	28.2	71.8	0.68	−	−	−	−	−	−
China (13)	−	−	−	22.6	77.4	<0.001	−	−	−
Istanbul (14)	−	−	−	−	−	−	66	34	0.002
Egypt (15)	−	−	−	−	−	−	71	29	<0.001

### Haplotypes analysis

The analysis of haplotypes revealed one block in linkage disequilibrium with D’ = 0.579 and r^2^ = 0.127, which was composed of two polymorphisms (rs1800477 and rs2740483). The analyses showed three possible allele combinations (CA, CG, and GA). However, the distribution of these haplotypes was similar among CAD patients and the control group (data not shown).

### *ABCA1* mRNA expression

**Figure 1:**
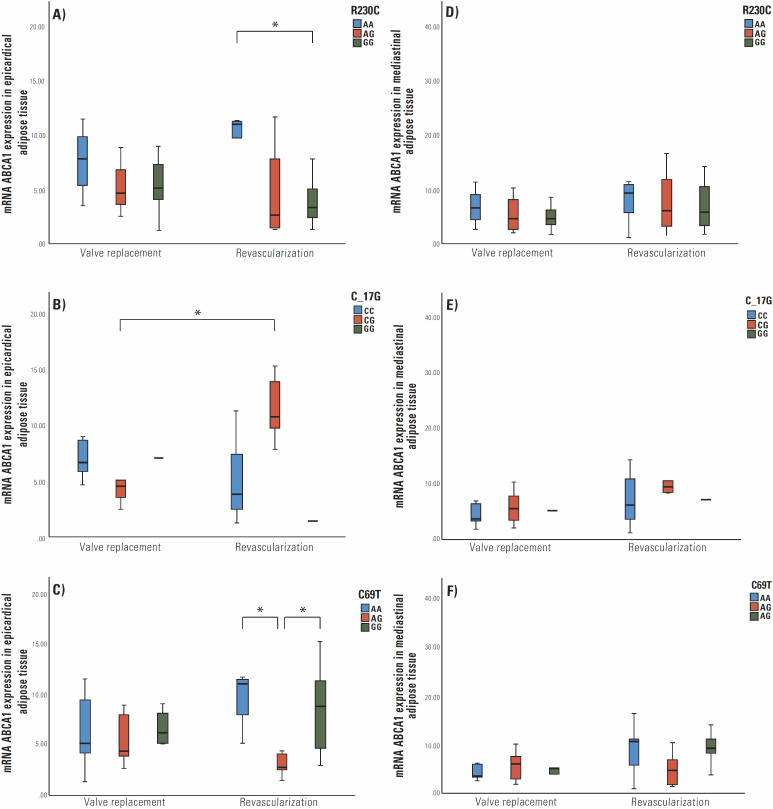
Differential expression of *ABCA1* normalized with *HPRT1* in epicardial adipose tissue (EAT) and mediastinal (MAT). (**A**) *ABCA1* expression in EAT in R230C genotypes; (**B**) *ABCA1* expression in EAT in C-17G; (**C**) *ABCA1* expression in EAT in C69T; (**D**) *ABCA1* expression in mediastinal adipose tissue in R230C; (**E**) *ABCA1* expression in MAT in C-17G; (**F**) *ABCA1* expression in MAT in C69T. p > 0.05*.

To determine the expression of *ABCA1* mRNA in the CAD and control groups, mRNA was quantified in epicardial and MAT. First, the expression of *ABCA1* was compared between all the revascularized groups and with valvular group in both types of tissues evaluated. However, we did not find significant differences (data not shown). Therefore, the expression results were stratified by genotypes of SNPs R230C, C-17G, and C69T. Figures 1A-C show the results obtained from EAT. SNP R230C expressions showed statistically significant under-expression in the GG genotype compared to AA in the revascularized patient group [11.01 (4.31-15.24) *vs.* 3.86 (2.47-12.50), p = 0.015] (Figure 1A).

The C-17G SNP showed no differences in the expression between the genotypes of the revascularized patients, but compared with the control group, the AG genotype in patients with CAD presented overexpression [12.02 (7.88-15.24) *vs.* 4.66 (7.88-15.24), p = 0.011] (Figure 1B). For the GG genotype, however, there was only one patient for allelic comparison, so we could not make conclusions about the expression of *ABCA1* for this SNP.

Finally, SNP C69T showed significant differences between the AA and AG genotypes and between the AG and GG genotypes in revascularized patients [11.01 (5.10-11.68) *vs.* 2.63 (1.35-12.50), p = 0.034; and 2.63 (1.35-12.50) *vs.* 8.79 (2.87-15.24), p = 0.010, respectively]. Figures 1D–F show the expressions according to genotype in MAT, which presented significant differences.

## DISCUSSION

The aim of this work was to evaluate polymorphisms and mRNA expression of the ABCA1 transporter in epicardial and MAT from patients with CAD. An increase in the R230C GG genotype was associated with decreased mRNA expression in EAT in patients with CAD. Importantly, we observed statistical differences in mRNA expression of *ABCA1* in only EAT from CAD patients. Polymorphisms in the *ABCA1* gene may induce several lipid transport defects and deficiencies in HDL cholesterol, leading to the appearance of atheromatous plaque. The significant differences in the R230C SNP in the overdominant model could suggest that the minor allele is a cardiovascular risk factor.

Previous reports on human embryonic kidney cell lines (Flp-In™-293) expressing the *ABCA1* C230 allele indicated a cholesterol efflux that is 27% lower than that observed in cells expressing the wild-type R230 allele (12). This is consistent with the decrease in mRNA expression observed in our results. In addition, it has been reported that the *ABCA1* polymorphism R230C (rs9282541) is almost exclusively present among indigenous peoples of the Americas and related populations. It has been associated with low HDL-c levels (13–15), type 2 diabetes mellitus (16), and obesity (17), which are factors associated with the formation of atheromatous plaque. An increased risk of death from CHD is associated with abnormally low HDL-C for cholesterol ranges both below and above 5.2 mmol/L (18).

An association has been reported between the *ABCA1* R230C polymorphism with hypoalphalipoproteinemia (19). Frikke-Schmidt and cols. found that approximately 10% of individuals with the lowest percentile of HDL cholesterol are heterozygous for mutations in *ABCA1* (20). These data agree with our results, but only for R230C polymorphisms for the CAD group, which had the lowest percentage of heterozygotes with low HDL-C levels and low mRNA expression in both adipose tissue and the mediastinum. However, we did not find a significant association with HDL-C levels. Previous studies on Mexican populations have reported low levels of HDL-C in the general population (16). In addition, patients with CAD take statins, which are known to affect HDL levels, which could explain the lack of association between disease and HDL-C levels.

For C-17G, we observed an increase in CC genotype with significant differences in all models analyzed, and there was higher expression of mRNA in CG in EAT in patients with revascularization. Little information exists on the C-17G SNP and its association with cardiovascular disease, but reports show that the major allele's prevalence could be associated with the development of atheromatous plaque (21,22). C-17G is located 17 bp upstream in the 5’-untranslated region (UTR) of the *ABCA1* gene in a possible transcription factor-binding and enhancing part. A possible molecular mechanism of action of this SNP is that mutations in the promoter sequence in the *ABCA1* gene cause changes in the affinity of Pol II for the promoter site, generating a decrease in mRNA, which could increase the risk of developing CAD. This agrees with our study since the prevalence of the CC genotype and the presence of the C allele were higher among patients with CAD, as was the mRNA expression.

We did not find significant differences between genotypes when quantifying *ABCA1* mRNA and stratifying according to genotypes, which could possibly be due to the sample size. However, when reviewing the AG expression of the CAD and control groups, there was overexpression in the CAD group. The reason could be that patients undergoing revascularization are receiving different pharmacological treatments, diets, and physical exercises that could promote overexpression to compensate for the damage caused by the formation of atheromatous plaque (23).

Finally, C69T was associated with the additive model but had lower mRNA expression in the CT genotype. Ergen and cols. (24) observed no significant differences in the distribution of C69T genotypes between study groups, but they found that the CC genotype was associated with increased MI, triacylglycerol, and VLDL cholesterol levels in patients compared to those with the CT genotype. Sheidina and cols. reported no association between C69T genotypes and male patients who had myocardial infarction before age 45 (25). Despite the different polymorphisms, our study had similar findings about the relationship between *ABCA1* polymorphisms and their effects on lipids levels.

Our results were similar to those of a previous study investigating the association of C-69T polymorphism with plasma lipid levels and CAD. They found no significant differences in the distribution of C-69T genotypes between study groups (26). The mRNA expression in the C69T SNP showed differences in the group of revascularized patients, with the heterozygous genotype presenting lower expression compared to the homozygous dominant and recessive genotypes. Although we found significant differences in the expression levels of *ABCA1* in the 3 SNPs studied, there was no association between lipid levels and the relative expression of mRNA. Although drugs probably affected the results, *ABCG1* also mediates cholesterol transport (27).

Reports show that ablation of *ABCA1* and *ABCG1* in macrophages *in vitro* resulted in substantial lipid accumulation. In contrast to deleting just *ABCA1, ABCG1* may thus compensate for the loss of *ABCA1* in macrophages. Therefore, to confirm this hypothesis, it is necessary to quantify the *ABCG1* mRNA as well. *ABCA1* mRNA expression did not present significant differences when between groups in MAT. This could be due to the atheromatous process in patients with CAD occurring in the coronary arteries, where the closest adipose tissue is the EAT. In addition, it has been reported that *ABCA1* mRNA expression is influenced by risk factors such as smoking, age, diet and obesity (28).

The genotype frequencies showed significant differences from those of other populations, which could be due to eating habits and physical activity. Differences in genetic load between populations could be a factor in the appearance of coronary disease. The variance between previous studies and our study might have been due to ethnic and racial differences, which might have influenced the results.

A limitation of the study design was that the control group had some valve disease due to the difficulty of obtaining fatty tissue samples from healthy subjects. In addition to the fact that patients with CAD underwent revascularization surgery, the vast majority were medicated and on strict diets, and lipid and glucose levels were altered. Therefore, the analyses to correlate profiles with genotypes could have abnormal results.

The age was also significantly different between groups. However, CAD usually occurs at ages older than 50 years, while for patients receiving valve replacement (the control group), age can vary considerably in the population since the etiology of the diseases is different. In addition, it is essential to study other *ABCA1* SNPs like rs2422493 (−565C>T), as well as *ABCG1* SNPs, and more patients should be included.

In conclusion, the results suggest that genotype GG of R230C and genotype CC of C-17G polymorphisms in the *ABCA1* gene are strongly associated with the development of CAD in Mexican patients. In addition, mRNA under-expression in the GG genotype in R230C is associated with CAD. Follow-up studies are necessary to better understand the role of candidate gene polymorphisms in the effects of mRNA expression levels on CAD.
